# The toxicity of coated silver nanoparticles to *Daphnia carinata* and trophic transfer from alga *Raphidocelis subcapitata*

**DOI:** 10.1371/journal.pone.0214398

**Published:** 2019-04-03

**Authors:** Sam Lekamge, Ana F. Miranda, Andrew S. Ball, Ravi Shukla, Dayanthi Nugegoda

**Affiliations:** 1 Ecotoxicology Research Group, School of Science, RMIT University, Bundoora, Victoria, Australia; 2 Centre for Environmental Sustainability and Remediation, School of Science, RMIT University, Bundoora, Victoria, Australia; 3 Nanobiotechnology Research Laboratory (NBRL), School of Science, RMIT University, Melbourne, Victoria, Australia; VIT University, INDIA

## Abstract

Nanoparticles (NPs) are causing threats to the environment. Silver NPs (AgNPs) are increasingly used in commercial products and may end up in freshwater ecosystems. The freshwater organisms are vulnerable due to water-borne and dietary exposure to AgNPs. Surface properties play an important role in the fate and behavior of AgNPs in the aquatic environment and their effects on organisms. However, effects of surface properties of AgNPs on organisms are poorly understood. In this study, we explored the effects of AgNPs coated with three different ligands; Tyrosine (T-AgNP), Epigallocatechin gallate (E-AgNP) and Curcumin (C-AgNP) in relation to the toxicity to a key aquatic organism; *Daphnia carinata*. The study focused on how coatings determine fate of NPs in the medium, mortality, feeding behaviour, bioaccumulation and trophic transfer from the freshwater alga, *Raphidocelis subcapitata* to daphnids. NP stability tests indicated that T-AgNPs were least stable in the ASTM daphnia medium while C-AgNPs were most stable. 48 h EC_50_ values of AgNPs to *D*. *carinata* were in the order of E-AgNP (19.37 μg L^-1^) > C-AgNP (21.37 μg L^-1^) > T-AgNP (49.74 μg L^-1^) while the 48 h EC_50_ value of Ag^+^ ions was 1.21 μg L^-1^. AgNP contaminated algae significantly decreased the feeding rates of daphnids. However, no significant differences were observed in feeding rates between algae contaminated with differently coated AgNPs. Trophic transfer studies showed that AgNPs were transferred from algae to daphnids. The bioacumulation of AgNPs in algae and the diet-borne bioaccumulation of AgNPs in daphnids varied for differently coated AgNPs. Bioaccumulation of C-AgNPs in algae was 1.5 time higher than T-AgNPs. However, the accumulation of T-AgNPs in daphnids via trophic transfer was 2.6 times higher than T-AgNPs. The knowledge generated from this study enhances the understanding of surface property dependent toxicity, bioaccumulation and trophic transfer of AgNPs in aquatic environments.

## Introduction

Engineered nanoparticles (ENPs) are man-made materials with a size range of 1 to 100 nm [1]. Global production of ENPs are increasing exponentially as they are widely being used in many applications such as healthcare, personal care, construction, energy, electronics, catalysts, packaging, textiles, environmental remediation and agriculture [2–4]. Despite their useful applications, the physicochemical characteristics that make NPs unique are causing possible threats to the health of the environment including humans. Unfortunately, understanding the implications of NPs have not kept pace with the advancements of nanotechnology and therefore, concerns are growing about their possible environmental health and safety risks among the scientific community, regulatory agencies and general public [1, 5–8]. There are 1800 plus nano-enabled consumer products in the market while silver nanoparticles (AgNPs) is the most frequently used nano-material (435 products) as reported by Vance, Kuiken [9]. Certain physicochemical, chemical and structural features of AgNPs are useful as an excellent antimicrobial agent [10–15] and in the fields of material science, chemistry and physics [16]. With increased usage in consumer products, large quantities of AgNPs end up in aquatic ecosystems posing a huge threat to aquatic organisms. Primary producers are vulnerable to water-borne exposure while higher organisms are affected by both water-borne and diet-borne exposure [17, 18]. Complete assessment of health and environmental impacts of engineered nanomaterials is not possible due to lack of nanotoxicity and ecotoxicity data and it will take many more years to produce actionable information leaving all concerned parties with little guidance [7]. Therefore, further studies are required to minimize the harm caused by NPs considering the diversity of NP characteristics [16, 19–22].

AgNPs may cause mechanical [23–25] and physiological [26, 27] damage. Some researchers claim that liberated ions from AgNPs are the only cause of toxicity to aquatic organisms [28, 29] while other studies indicate that particles are the major cause of toxicity [30, 31]. The toxicity of NPs are compared to the toxicity of the counterpart bulk material, usually metal salts to test this hypothesis [32]. Surface coatings of NPs have a strong influence on their physicochemical properties and can also influence the toxicity to organisms [33]. Though there are studies on effects of coated NPs, further studies are required due to addition of new coating materials, different views on effects of coatings and to protect native freshwater species. Zhao and Wang [34] found AgNPs coated with sodium dodecylbenzene sulfonate caused highest toxicity (48 h LC_50_: 1.1 μg L^-1^) to *Daphnia magna* followed by polyvinylpyrrolidone (PVP) (48 h LC_50_: 2.0 μg L^-1^) and lactate-coated AgNPs (48 h LC_50_: 28.7 μg L^-1^). Silva, Pokhrel [33] studied the toxicity of three types of organo-coated AgNPs to *D*. *magna*. They found that the branched polyethyleneimine-coated AgNPs (48 h LC_50_: 0.41μg L^-1^) were most toxic followed by citrate (48 h LC_50_: 2.88 μg L^-1^) and PVP-coated AgNPs (48 h LC_50_: 4.79 μg L^-1^). Newton, Puppala [35] exposed *D*. *magna* to three types of coated AgNPs for 48 h in two different media. They found gum Arabic-coated AgNPs (48 h LC_50_: 2.14–3.48 μg L^-1^) were most toxic followed by polyethylene glycol (48 h LC_50_: 2.27–13.08 μg L^-1^) and PVP-coated AgNPs (48 h LC_50_: 14.04–14.81 μg L^-1^).

NPs can be bioaccumulated and transferred from one trophic level to another through the food chain [36–38]. Trophic transfer studies allow us to differentiate the importance of different exposure routes which is useful in risk analysis. Algae are primary producers of energy as a food source and any impacts at this level may affect the health of organisms at higher trophic levels [39, 40]. Several studies have shown that food is the major source of AgNP accumulation in *D*. *magna* [39, 41]. Zhao and Wang [41] found that AgNPs were more efficiently assimilated in daphnids and was more difficult to depurate when NPs were ingested through the dietary intake than water-borne exposure. McTeer, Dean [42] observed trophic transfer of NPs to *D*. *magna* from AgNP treated alga *Chlamydomonas reinhardtii*. Chae and An [43] saw trophic transfer of Ag nanowires (AgNWs) from *C*. *reinhardtii* to *D*. *magna* and then to the fish *Danio rerio*. Lee, Yoon [44] observed trophic transfer of gold NPs (AuNPs) to *D*. *magna* from *C*. *reinhardtii* and *Euglena gracilis*. Bouldin, Ingle [45] observed transfer of Carboxyl quantum dots (QD) from QD-exposed alga *Raphidocelis subcapitata* to *Ceriodaphnia dubia*. Elsewhere, trophic transfer of ENPs were demonstrated from algae to mussels [46], daphnids to zebrafish [47] and biofilms to snails [48]. Exposure to AgNPs and Ag^+^ ions contaminated algae also cause changes in feeding behaviour in daphnids. McTeer, Dean [42] also reported a significant reduction in feeding when daphnids were fed with AgNP and Ag^+^ ion contaminated algae compared to untreated algae. Zhu, Chang [49] observed dose dependent reductions in ingestion and filtration rate when *D*. *magna* was exposed to TiO_2_ NPs.

In this study, we studied the toxicity of AgNPs coated with different organic ligands; Tyrosine (T-AgNP), Curcurmin (C-AgNP) and Epigallocatechin gallate (E-AgNP) to the freshwater filter-feeding cladoceran, *Daphnia carinata*. In addition, the effects of associated Ag with alga on daphnid feeding behaviour and trophic transfer from the alga diet to daphnids were investigated. Tyrosine, Curcurmin and Epigallocatechin gallate have different number of phenol structures and classified as mono-phenol, bi-phenol and poly-phenols respectively. Since they are organic compounds, their usage is considered as a green and ecofriendly approach to produce NPs while they are biocompatible which is a useful characteristic in therapeutic applications [50]. These coatings are used to produce NPs for different applications, mainly being found in medical applications [50–54]. Though there are studies on the effects of AgNPs with most commonly used coatings, studies on organic coatings used in this study are lacking. *Daphnia sp*. is one of the most sensitive species used in aquatic toxicological studies and is recommended as a model test organism by international agencies. The most common species used for studies is *D*. *magna* which is considered as an invasive species in some parts of the world. Therefore, it is required to improve test species and protocols to better reflect species sensitivity in different ecosystems [55], for environmental risk assessment and to protect native species. Also, daphnids represent the bottom level of the freshwater food chain and any qualitative or quantitative effect to the population will affect higher organisms [16]. To the best of our knowledge, this is the first study where the effects of differently coated NPs were studied against *D*. *carinata* while other studies so far have used *D*. *magna*. Also, the coating materials are quite novel and have not being used in similar kind of studies.

## Materials and methods

### 2.1 Preparation of NPs

L-tyrosine, epigallocatechin gallate (EGCG) and curcumin (Fig A1, A2 and A3 in S1 File) were obtained from Sigma-Aldrich. NP synthesis was performed in-house as previously described by Selvakannan, Swami [53]. Briefly, 10 mL of 1 mM Ag_2_SO_4_ solution was mixed with 10 mL of 1 mM aqueous solution of Tyrosine, EGCG and Curcumin and diluted to 100 mL with MilliQ water. To each solution, 1 mL of 100 mM KOH solution was added, and the mixture was allowed to boil until the colour of the solution turned yellow which indicates NP formation. The AgNP solutions were allowed to age for 1 day and then concentrated by rotary evaporation. The solutions were then dialyzed for 48 h in a dialysis tube (MWCO: 3 kDa) which was submerged in copious amounts of MilliQ water with stirring to remove any uncoordinated silver ions, excess KOH and unbound coating materials. Water was replaced twice with fresh MilliQ water after 6 and 24 h. The dialyzed AgNP solutions were stored in the dark. Each type of NP (0.1 mL) was acid digested with ultra-pure grade 70% HNO_3_ (Thermo Fisher Scientific, NSW, Australia) on a heating block at 105 ^o^C for 12 h. The digested samples were then diluted with MilliQ water and the silver (Ag^+^ ions) concentrations were measured by inductively coupled plasma mass spectrometry (ICP-MS) (7700x, Agilent Technologies).

### 2.2 Characterization of NPs

The zeta potential and hydrodynamic diameter (HDD) of the AgNPs were measured using a folded capillary cell and glass cuvette, respectively, on a Zetasizer (Dynamic light scattering; Malvern Zetasizer Nano series, NanoZS). The particles were drop cast on carbon copper grids and images were taken using a transmission electron microscope (TEM) operated at an accelerating voltage of 100 kV (TEM, JEOL 1010) equipped with a Gatan imaging system. The mean core size of NPs was determined from TEM images using ImageJ software. The surface plasmon resonance (SPR) was recorded as absorbance spectra using a UV-visible spectrophotometer (Varian Cary 50) operated at a resolution of 2 nm, from 200 to 800 nm, using a quartz cuvette with a path length of 1 cm. Concentrations of stock solutions were measured with ICP-MS (7700x, Agilent Technologies) after acid digestion with concentrated HNO_3_.

### 2.3 Culturing organisms

*R*. *subcapitata* pure cultures were maintained in the lab and the algal cells were inoculated in the MLA medium to prepare the algae stock cultures. MLA medium was prepared as described by Bolch and Blackburn [56] with slight modifications. Briefly, concentrate nutrient stock solutions were prepared by filter sterilization and the medium was prepared in MilliQ water by autoclaving for 15 min at 121°C. The algae stock cultures were maintained axenically as per the OECD guidelines [57]. Cultures were aerated and incubated in a light-temperature controlled chamber at 23 ± 1°C under continuous illumination (6000 lux). White fluorescent tubes were used as the light source while the light intensity in the test setup was measured using a LI-COR light meter (model LI-189). The pH was maintained at 7.5 ± 0.5. Algal cells were counted periodically with an automated cell counter (TC20, Bio-Rad Laboratories, Hercules, CA) to make sure the culture is at exponential growth stage when it is treated with NPs for the daphnid feeding and bioaccumulation test. A widespread *Daphnia* species in Australia, *D*. *carinata* was used to assess the acute toxicity, feeding behaviour and bioaccumulation experiments. ASTM standard medium [58] was used for stock culturing and in all experiments. Daphnid stock culture was maintained at 20 ± 1°C with 16:8 h light and dark photoperiod to obtain neonatal cladocerans. They were fed with *R*. *subcapitata* (5 × 10^5^ cells mL^-1^), a highly suitable nutrient source for daphnids. The medium was aerated to make it saturated with oxygen before addition of daphnids. The medium was renewed three times a week, and the pH was maintained at 7.5 ± 0.2 throughout and cultures were maintained in glass beakers which were loosely covered to prevent any contamination and evaporation.

### 2.4 AgNP temporal stability and dissolution in the test medium

The stability of AgNPs in the ASTM medium and MilliQ water was investigated as previously described by Tejamaya, Römer [59] with some modifications. Nanoparticle solutions of 5,000 μg L^-1^ were incubated in glass vials for 24 h at similar environmental conditions to the daphnid acute toxicity test. The SPR, HDD and zeta potential of the suspensions were investigated, and pH was monitored. The release of Ag^+^ ions from NPs was investigated in the ASTM medium and MilliQ water over 24 h as previously described by Xia, Kovochich [60] with some modifications. Briefly, 1 mL from each NP suspension was extracted into Eppendorf tubes and centrifuged at 21,000 rpm for 15 min (Sigma 3-KL centrifuge). Supernatant (0.75 mL) from carefully removed tubes was transferred to 15 mL tubes, acidified with HNO_3_ and diluted with MilliQ water. The Ag^+^ ion concentrations were measured by ICP-MS (7700x, Agilent Technologies). The samples need to be in ionic form prior to entering the mass analyser in order to be detected. The coated NPs were not detectable as validated by independent experiments by using undigested NPs. Therefore, the influence of NPs which may be present in the supernatant to the readings was insignificant.

### 2.5 Acute toxicity bioassay with *D*. *carinata*

AgNP test solutions of relevant nominal concentrations for the acute test were prepared based on ICP-MS results just before the test began by sonicating and dispersing relevant volumes of stock solutions in daphnid culture medium. Ionic silver stock solution was prepared by dissolving Ag_2_SO_4_ in culture medium followed by ICP-MS analysis and required concentrations for acute tests were prepared by dissolving relevant volumes from the stock solution in daphnid culture medium. Controls contained only the culture medium. Semi-static renewal acute toxicity tests were performed according to the OECD standard procedure [61]. Less than 24 h old third brood progeny was used for experiments. Daphnid neonates were exposed to seven different concentrations of each test substance for 48 h. Test concentrations were chosen based on results obtained from range finding tests. Concentrations employed for the tests were in the range of 10.0–40.0 μg L^-1^ for E-AgNP, 30.0–90.0 μg L^-1^ for T-AgNP, 10.0–35.0 μg L^-1^ for C-AgNP and 0.6–1.8 μg L^-1^ for Ag^+^ ions. For each concentration, 5 neonates (age: < 24 h) were placed in a 35 mL glass vial containing 15 mL of test solution. The test solution was renewed after 24 h and all experiments were conducted in quadruplicate (n = 4). Daphnids were not fed during the 48 h time period. The tests were conducted at 20 ± 1°C with 16:8 h light and dark photoperiod, pH was maintained at 7.5 ± 0.2 while the dissolved oxygen concentration in all the test solutions exceeded 3 mg L^−1^. Immobilization was recorded after 24 and 48 h while they were considered immobile if they couldn’t move within 15 s of gentle agitation of the test container. The toxicity tests were considered valid if the mortality was not >10% in the control.

### 2.6 Feeding analysis and AgNP trophic transfer

Algae feeding experiment was conducted as described by McTeer, Dean [42] and Grintzalis, Dai [62] with some modifications. Cell density of the algae culture which was in exponential growth stage was adjusted to 5 × 10^4^ cells mL^-1^. Algae cells were propagated in a 1 L Erlenmeyer flask containing 500 mL algae culture in the presence of particles (50 μg L^-1^) or absence (blank control) in triplicate. The flasks were incubated as per the OECD guideline [57] on orbital shakers (OM6, RATEK, Aus) at 100 rpm under the same environmental conditions used for algae culturing. Algal cell counts of each flask were taken with the automated cell counter and the specific algal growth rate of all treatments were calculated for each day. Algae cells were fed to daphnids after 6 days when the cells had reached stationary growth phase. Since the cells were no longer dividing, number of cells consumed could accurately be measured. A volume of 350 mL from each culture was centrifuged (3000 *g*, 5 min, 20°C) and washed three times with MilliQ water to remove any loosely adsorbed contaminants from algae. The algae were then resuspended in ASTM medium in a 50 mL Falcon tube as it contained approximately 5 × 10^6^ cells mL^-1^ and stored at 4°C. Feeding experiments were carried out under similar environmental conditions used for the acute toxicity test in 100 mL glass beakers in triplicate with 50 mL ASTM medium in each beaker. Daphnids (15, age: < 24 h) were placed in each beaker and fed with AgNP treated and untreated algae for 5 days. Daphnids were transferred to fresh media after every 24 h and the starting and final algal cell numbers were determined with the automated cell counter. The solutions were stirred on a magnetic stirrer for 20 s before samples were taken for cell counting. The 5 day feeding phase was followed by a 3 day depuration phase in fresh medium where daphnids were fed with unexposed algae. Then, daphnids were oven dried at 60°C for 48 h and acid digested on a block heater for 6 h at 100°C with 70% HNO_3_ and 30% H_2_O_2_. Digested samples were diluted with MilliQ water and analysed using ICP-MS. A volume of 50 mL from each remaining culture was harvested by centrifugation (3000 *g*, 5 min) and washed three times with MilliQ water. The resulting algal pellets were oven dried at 60°C for 48 h and acid digested separately as described above. The digested samples (0.5 mL) were diluted with MilliQ water and the silver (Ag^+^ ions) concentrations were measured using ICP-MS (7700X, Agilent Technologies).

### 2.7 Data analysis

The 24 and 48 h EC_50_ values and their associated 95% confidence intervals (95% CI) were calculated using the TOXRat software (TOXRat solutions GmbH, version 3.0). The statistical method used was the probit analysis using linear maximum likelihood regression. The daily feeding rate was determined by dividing the number of algal cells consumed by the number of daphnids that were alive in each beaker. Trophic transfer was quantitatively determined by calculating the transfer of Ag to a daphnid from 10^5^ algal cells consumed. For each dataset, mean and SD are presented and data were considered statistically significantly different at *P* < 0.05. All data were normally distributed according to Shapiro-Wilk test. Differences between treatments were analysed using one-way analysis of variance (ANOVA). When significant differences were detected at a 95% level of confidence, the Tukey’s multiple comparisons were applied. ANOVA was performed using Sigmaplot statistics version 13. Differences of treatments over time for repeated measures were determined by 2-factor ANOVA followed by Holm-Sidak method.

## Results and discussion

### 3.1 Particle characterization

**Fig 1:**
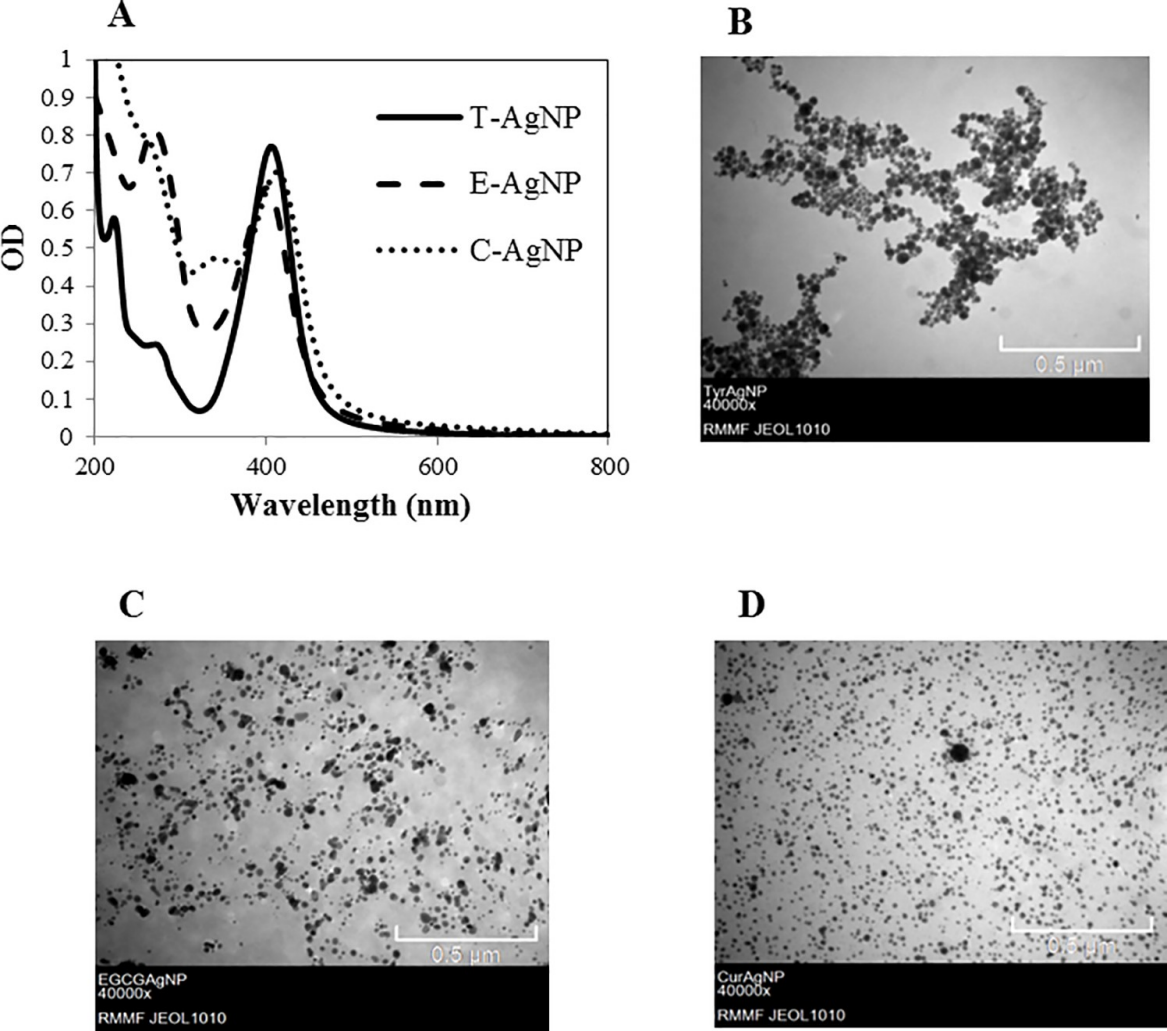
The SPR spectra and TEM images of AgNPs. (A) The SPR of AgNPs in MilliQ water. (B) T-AgNPs. (C) E-AgNPs. (D) C-AgNPs.

Strong, well defined absorptions peaks at 406 (T-AgNP), 400 (E-AgNP) and 414 nm (C-AgNP) in the SPR curves (Fig 1A) indicate the presence of AgNPs [53, 63–66]; these peaks were absent in controls which included media, pure coating material and coating material plus KOH. The presence of NPs was further confirmed by TEM analysis (Fig 1B, 1C and 1D) which revealed spherical shaped NPs which were reasonably uniform in size. Intensity weighted mean HDD and zeta potential values in MilliQ water and core sizes of AgNPs are shown in Table 1. Zeta potential values below -30 mV suggest that organic molecules formed strong coatings on the AgNPs [50]. The secondary peaks of SPR spectra at 272 (T-AgNP), 270 (E-AgNP) and 256 nm (C-AgNP) are due to the Ag-bound tyrosine [53, 67], EGCG [68] and curcumin molecules [69] respectively. The concentrations of T-AgNP, E-AgNP, C-AgNP and Ag_2_SO_4_ stock solutions as measured by ICP-MS were 45.59, 48.84, 42.66 and 10.52 mgL^-1^ respectively.

**Table 1:** Summary of AgNP sizes and zeta potential in MilliQ water. Standard deviations (± SD) are from triplicates.

	T-AgNP	E-AgNP	C-AgNP
HDD (nm)	51.58 ± 0.55	40.06 ± 1.50	36.37 ± 0.58
TEM average size (r.nm)	10.56 ± 2.27	9.27 ± 1.29	13.68 ± 0.76
Zeta potential (mV)	-42.13 ± 0.33	-38.93 ± 1.37	-44.65 ± 1.65

### 3.2 Stability of coated AgNPs in ASTM medium

**Fig 2:**
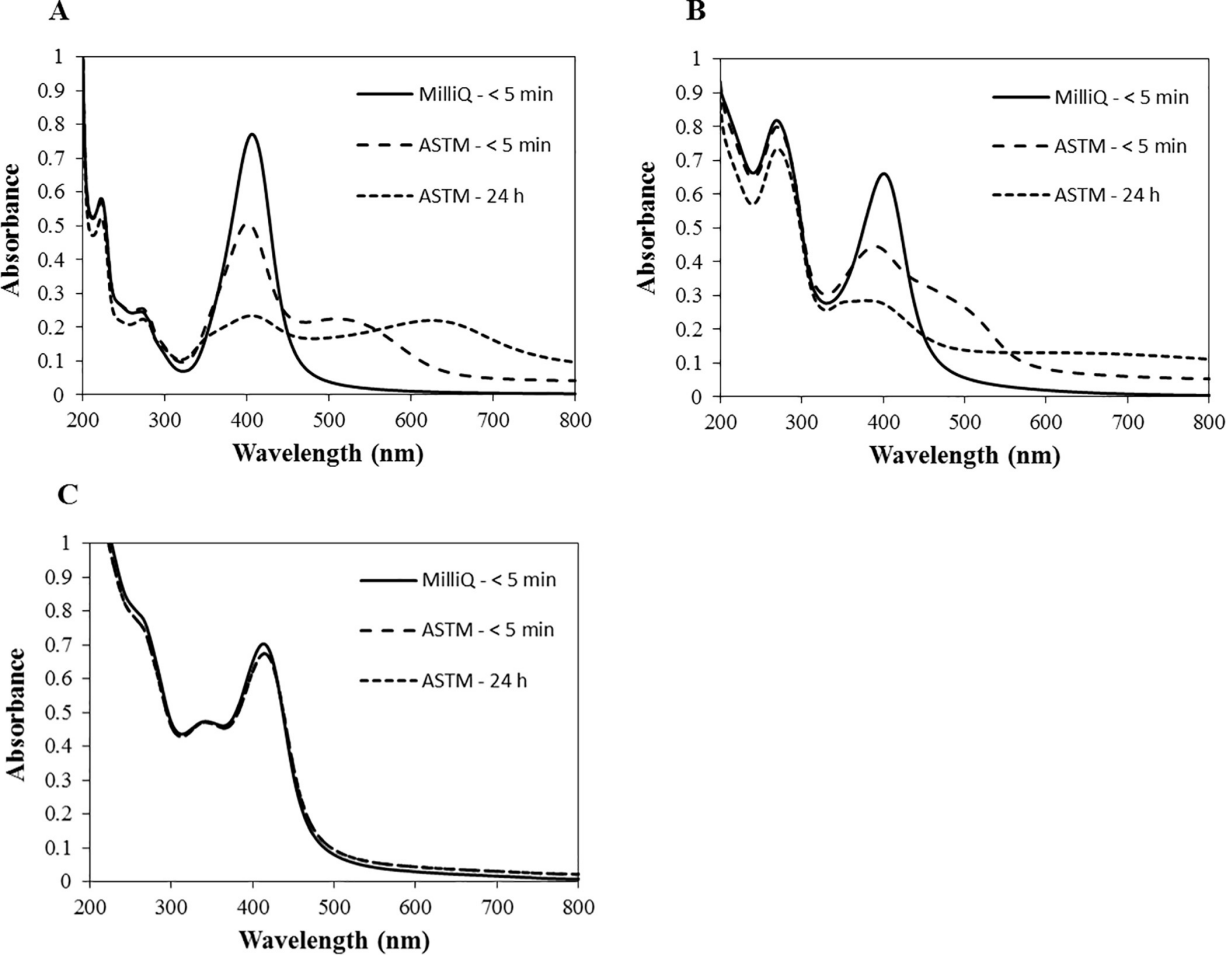
The SPR of AgNPs in MilliQ water and ASTM medium. (A) T-AgNPs. (B) E-AgNPs. (C) C-AgNPs.

Suspensions of AgNPs in MilliQ water were initially yellowish in colour with absorption peaks at 406, 400 and 414 nm for T-AgNPs, E-AgNPs and C-AgNPs respectively. Once the AgNPs were dispersed in ASTM medium, visual observation revealed that the T-AgNP and E-AgNP suspensions turned to yellowish brown within minutes, but the yellowish colour of C-AgNP suspension was stable even after 24 h. SPR analysis revealed a larger decrease in the absorption peaks of T-AgNPs (34 & 70%) and E-AgNPs (33 & 57%) after 5 min and after 24 h respectively in comparison to the initial relevant absorption peaks of AgNPs in the MilliQ water measured after 5 min (Fig 2A and 2B). In contrast, the decrease of the absorption peak was 4 & 5% for C-AgNPs after 5 min and 24 h (Fig 2C). In addition, the SPR bands of T-AgNPs and E-AgNPs significantly broadened which was not observed for C-AgNPs. However, the SPR bands of AgNPs didn’t change considerably in the MilliQ water (Fig B1, B2 and B3 in S1 File). In the ASTM medium, the percentage change of dissolution after 24 h in comparison to the dissolution after first 5 min was 0.06, 0.04 and 1.51% for T-AgNPs, E-AgNPs and C-AgNPs respectively while it was 0.04, 0.36 and 4.39%, respectively in MilliQ water. The mean HDD of T-AgNPs and E-AgNPs in the ASTM medium increased approximately by 2.7 and 4.7 times respectively after 5 min and by 9 and 8 times after 24 h. However, the mean HDD of C-AgNPs did not change considerably (Table 2). In comparison, none of the AgNPs showed considerable change in HDD in the MilliQ water (Table A in S1 File). The percentage intensity distribution of particle size (weighted according to the scattering intensity of each particle fraction) in media revealed that approximately 63, 79 and 87% of aggregates of T-AgNPs, E-AgNPs and C-AgNPs remained at the sub-micron level after 24 h. In comparison to the zeta potential of NPs in the MilliQ water (Table A in S1 File), it sharply increased by 13.4, 23.9 and 24.3 mV of T-AgNPs, E-AgNPs and C-AgNPs respectively after 5 min but did not change considerably thereafter (Table 2). The polydispersity index (PdI) of T-AgNPs and E-AgNPs slightly increased in both ASTM medium (Table 2) and MilliQ water (Table A in S1 File).

**Table 2:** HDD, zeta potential and PdI of AgNPs measured after 5 min and 24 h. AgNPs were dispersed in the ASTM media at Ag concentration of 5,000 μg L^-1^. Standard deviations (± SD) are from triplicates.

Substance	Medium	HDD (nm)		Zeta Potential (mV)		PdI	
		< 5 min	24 h	< 5 min	24 h	< 5 min	24 h
**T-AgNP**	**ASTM**	116.3 ± 6.4	394.1 ± 90.0	˗ 26.3 ± 1.6	˗ 24.0 ± 0.9	0.35 ± 0.10	0.63 ± 0.08
**E-AgNP**	**ASTM**	170.8 ± 33.8	290.4 ± 46.6	˗ 22.7 ± 3.1	˗ 22.5 ± 1.3	0.45 ± 0.04	0.55 ± 0.03
**C-AgNP**	**ASTM**	44.2 ± 8.6	49.7 ± 12.1	˗ 24.2 ± 1.4	˗ 23.3 ± 0.7	0.45 ± 0.04	0.46 ± 0.01

A broader absorbance peak, a large background signal and increased HDD indicate aggregation of NPs [59, 70]. As per the results, T-AgNPs and E-AgNPs aggregated rapidly in ASTM medium in comparison to C-AgNPs. Aggregation of NPs depends on particle concentration, pH, ionic strength, ionic composition, concentration and composition of natural organic matter, and other characteristics of the aqueous media [71, 72]. The observed differences in aggregation among coated AgNPs may be due to different coating materials since other characteristics of AgNPs such as initial HDD, shape and zeta potential are not significantly different (*p* > 0.05) [73, 74]. Also, all NPs showed no signs of aggregation in MilliQ water within the same time duration. The observed higher aggregation of AgNPs in ASTM medium in comparison to the MilliQ water could be attributed to the higher ionic strength of the medium. Decreased zeta potential values in ASTM medium with high ionic strength compared to MilliQ water with relatively low ionic strength are in accordance with the classical colloid theory [75, 76]. A reduction of the thickness of the diffuse double layer with increased ionic strength allows for the attractive van der Waals interactions to dominate and increases the particle-particle interaction resulting in increased aggregation [77, 78]. In general, the dissolution of AgNPs was higher in MilliQ water than in the ASTM medium. Köser, Engelke [79] and Levard, Mitra [80] reported that the percentage dissolution of AgNPs correlates to the Cl^-^ ion content (Cl/Ag ratio) in the medium which was contradictory to our results. This may be due to the precipitation of Ag ions by halides (Cl^-^) in the medium [81] which is not expected in the MilliQ water. Halides may reduce the exposure of organisms to any free ions released from NPs. Also, the size of the AgNPs is one of deciding factors for the release of Ag^+^ ions [82]. Small size particles have high surface to volume ratios and therefore, more atoms on the surface come into contact with oxidants in comparison to larger particles [83]. Aggregation of NPs results in larger particles with increased HDD reducing the surface area of particles available to release free Ag ions [34, 84]. Higher dissolution of AgNPs in MilliQ water than in ASTM medium may also be due to the less aggregation of AgNPs. The observed higher dissolution of C-AgNPs in both ASTM medium and MilliQ water than T-AgNPs and E-AgNPs is attributed to the less aggregation of C-AgNPs, while dissolution depends on several other factors [85].

Aggregation, dissolution and change in NP characteristics such as HDD as observed in this study may influence the bioavailability of NPs and hence, play an important role in determining toxicity [59, 86–88]. Also, culture medium impacted the behaviour and properties of AgNPs which may ultimately lead to various toxicological responses [89]. This study shows that the type of NP coating and medium significantly influence the degree of aggregation and the behaviour of AgNPs, which are required to consider in environmental risk assessment [90].

### 3.3 Acute toxicity of AgNPs to *D*. *carinata*

Evaluation of acute toxicity is crucial in environmental risk assessment of NPs in protecting the organisms and setting up water quality guidelines. According to the results in this study, the toxicity of AgNPs and Ag^+^ ions correlate with the concentration while the toxicity of Ag^+^ ions is significantly higher than AgNPs as per the 48 h EC_50_ values (Table 3). Among differently coated AgNPs, E-AgNPs showed the highest toxicity, but it was approximately 16 times less toxic compared to Ag^+^ ions. There was no much difference in toxicity between E-AgNPs and C-AgNPs (48 h EC_50_: 19.3 & 21.3 μg L^-1^), but T-AgNP was almost 2.5 times less toxic (48 h EC_50_: 49.7 μg L^-1^).

**Table 3:** 48 h EC_50, 20, 10_ values of *D. carinata* exposed to T-AgNP, E-AgNP, C-AgNP and Ag^+^ ions in the ASTM medium.

Substance	EC_50_			EC_20_			EC_10_		
	μg L^-1^	95% CI		μg L^-1^	95% CI		μg L^-1^	95% CI	
Ag^+^ ions	1.21	1.12	1.3	0.96	0.84	1.05	0.85	0.72	0.95
T-AgNP	49.74	45.4	53.86	38.7	33.31	42.76	33.94	28.06	38.27
E-AgNP	19.37	16.86	21.79	12.91	10.08	15.11	10.45	7.58	12.68
C-AgNP	21.37	19.41	23.26	16.88	14.33	18.7	14.92	12.09	16.87

NP size, type of coating, shape, charge are some major factors that influence toxicity [26, 33–35, 84, 91]. The shape of all NPs is spherical, and the initial size and charge do not significantly differ (*p* > 0.05) which may not explain the observed differences in acute toxicity. Therefore, the difference in toxicity is presumed to be the effects of different coating materials which reaffirm that different types of coating of NPs do have different effects on toxicity of NPs. T-AgNPs were least stable in the ASTM medium exhibiting the highest aggregation and settling of particles. Thus, the reduced toxicity of T-AgNPs compared to the other two types could be due to the lower bioavailability of NPs [71, 92]. However, the toxicity of E-AgNPs was similar to that of C-AgNPs which showed less aggregation and high dissolution compared with E-AgNPs. Therefore, the observed difference in toxicity could be a result of several factors which can’t be exclusively explained from the results of this study. Several previous studies have assessed the acute toxicity of AgNPs to *Daphnia sp*. and the reported EC_50_ values fall in the range of 0.26 to 236.3 μg L^-1^ for AgNPs and 0.16 to 12.9 μg L^-1^ for Ag^+^ ions [19, 29, 93–96]. The toxicity of coated AgNPs in this study is comparatively less than the values reported by majority of studies for other coated AgNPs. The broad range of toxicity values among published studies can be explained by different test scenarios and particle characteristics [84, 97]. *D*. *magna* has been the preferred species in many previous studies while this study used *D*. *carinata*.

Many research findings support the idea that the toxicity comes exclusively from Ag^+^ ions as the main source of toxicity [35, 98, 99]. Ag^+^ could prevent the absorption of Na across the membranes of gills by inhibiting the Na^+^, K^+^-ATPase activity and this could lead to ionoregulatory failure causing death [100]. The significantly lower EC_50_ value for Ag^+^ in comparison to NPs shows that the ionic silver is much more toxic than AgNPs. Coatings control the release of ions from AgNPs to the surrounding medium [34, 98]. As per the percentage dissolution of AgNPs, the computed concentrations of dissolved fractions of Ag^+^ ions from T-AgNPs, E-AgNPs and C-AgNPs at EC_50_ concentrations in the ASTM medium were 0.21, 0.13 and 1.06 μg L^-1^ respectively which were below the 48 h EC_50_ value of Ag^+^ ions. The toxicity may therefore not exclusively come from released Ag^+^ ions from AgNPs; NPs may have other toxic effects such as generation of reactive oxygen species (ROS) causing oxidative stress [26, 93, 101, 102], attachment to daphnid’s body surface or appendages leading to physical impairment and behavioral changes [103].

### 3.4 Feeding behaviour of *D*. *carinata*

**Fig 3:**
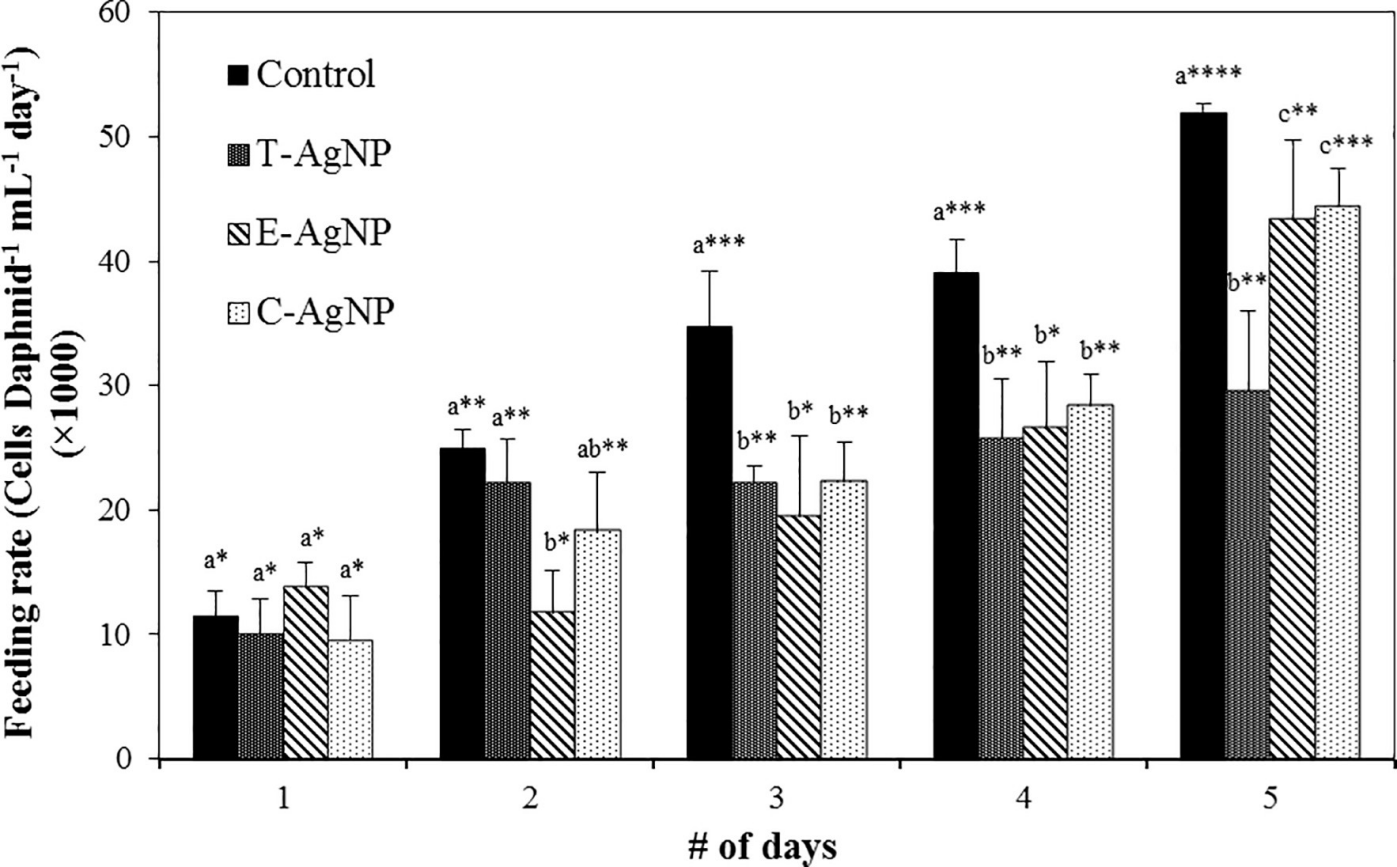
Algal feeding rates of *D*. *carinata* over a 5 d feeding period exposed to *R*. *subcapitata* cells that were treated with 50 μg L^-1^ concentrations of AgNPs. Data are mean ± SD from three independent experiments, each with 15 daphnids. The error bars indicate the SD (*p* < 0.05, *n* = 3). The *p*-values for multiple pairwise comparisons were obtained from two-way ANOVA followed by Holm-Sidak method using Sigmaplot. Letter sign denotes comparison of *p*-values of feeding rates for each day separately while the * sign denotes comparison of *p*-values of feeding rates over 5 days. Treatments that do not share lowercase letters or number of * signs are significantly different.

Daphnid feeding rate significantly increased from day 1 to 5 for all treated algae and in controls due to higher food consumption with ageing (Fig 3). The mean 5 day feeding rate was highest in the control followed by C-AgNP, E-AgNP and T-AgNP treated algae fed daphnids. Compared with the control, there was no significant deviation (*p* > 0.05) in feeding rate in the day 1 while only the feeding rate of E-AgNP treated algae was significantly different in the day 2. In contrast, the feeding rates of AgNP treated algae were significantly different to the relevant control from day 3 to 5 (*p* < 0.05). When compared with each treatment group, Daphnid feeding rates of algae treated with E-AgNPs in day 2 and T-AgNPs in day 5 were significantly different from other two types (*p* < 0.05). However, a consistent difference was not observed and therefore, the data obtained is not sufficient to prove any significant variations of feeding rates among daphnids based on algae treated with AgNPs with different coatings. Besides, less favoritism for untreated algae compared to treated algae shows that AgNPs have some effect on feeding behaviour. Previous studies also have shown that the feeding rate of daphnids became depressed when contaminants were associated with algae [104–106]. McTeer, Dean [42] observed a reduction in feeding rates when daphnids were fed with algae treated with polymer-coated AgNPs. Nutritional characteristics of all algae samples were similar and therefore, they emphasized that the feeding reduction was due to Ag toxicity but not due to the nutritional quality of the algae diet. Zhao and Wang [93] speculated that the reduced feeding rate was due to accumulation of NPs in the gut or due to higher sedimentation of contaminated algae to the bottom of the vessel causing less availability for filter feeding.

Inputs of energy from food is critically important for population growth and survival and therefore, the feeding rate in primary consumers like *Daphnia sp*. could have profound implications at the population level [104]. Feeding inhibition may cause reduction of growth, targeted inhibition of internal organs and reproduction of daphnids [93, 107–109]. It may also have effects on water clarity, altered nutrient regeneration rates, population size of predators [110] and elevated phytoplankton biomass due to reduced grazing [111]. Therefore, the observed feeding inhibition of algae associated with NPs by daphnids in this study is a cause of concern and should be considered in assessing aquatic NP pollution.

### 3.5 Trophic transfer of AgNPs from *R*. *subcapitata* to *D*. *carinata*

**Fig 4:**
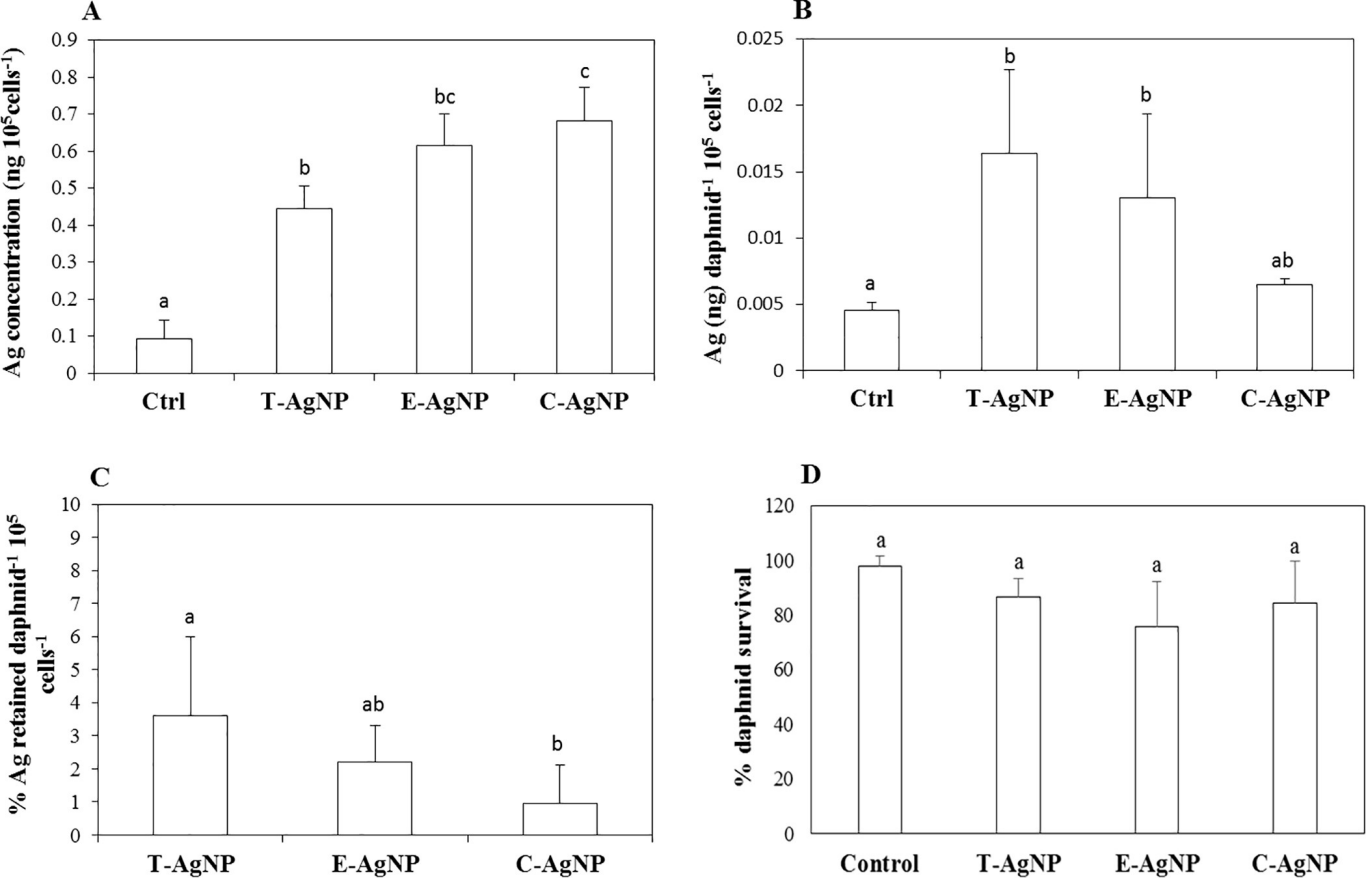
Bioaccumulation and trophic transfer of AgNPs. (A) Elemental Ag content per 10^5^ *R*. *subcapitata* cells measured after 5 days of growth in 50 μg L^-1^ concentrations of AgNPs or with no Ag treatment (control). (B) Elemental Ag content (per daphnid). (C) Percentage Ag retained (per daphnid). (D) Percentage survival of algae-fed *D*. *carinata* after 5 days of exposure to *R*. *subcapitata* cells that were grown in 50 μg L^-1^ or no Ag (control). The error bars indicate the SD (*p* < 0.05, *n* = 3). The *p*-values were obtained from one-way ANOVA followed by Tukey test using Sigmaplot. Treatments that do not share lowercase letters are significantly different.

The mean AgNP accumulation in algae treated with 50 μg L^-1^ C-AgNP (0.68 ± 0.09 ng 10^5^ cells^-1^), E-AgNP (0.61 ± 0.08 ng 10^5^ cells^-1^) and T-AgNP (0.44 ± 0.06 ng 10^5^ cells^-1^) concentration was significantly different to the control (0.09 ± 0.04 ng 10^5^ cells^-1^) (Fig 4A). C-AgNP accumulation in algae was significantly higher than T-AgNPs. However, the Ag accumulation profile in algae does not mirror the Ag accumulation profiles in daphnids. The mean Ag content in daphnids treated with T-AgNPs (0.016 ± 0.006 ng daph^-1^ 10^5^ cells^-1^) was significantly higher than the Ag content in daphnids treated with C-AgNPs (0.006 ± 0.0004 ng daph^-1^ 10^5^ cells^-1^) (Fig 4B). Also, Ag content in daphnids treated with T-AgNPs and E-AgNPs was significantly higher than the control. Though the T-AgNP treated algae accumulated lowest amount of NPs, it led to the highest mean percentage of Ag retention (3.6%) in daphnids followed by E-AgNP (2.1%) and C-AgNP treated algae (0.95%) (Fig 4C). In contrast, McTeer, Dean [42] found metal accumulation profiles in *D*. *magna* from trophic transfer correlated with the metal accumulation profiles in algae treated with ZnNPs. The Ag bioaccumulation in daphnids did not correlate with the daphnid survival percentage (Fig 4D) where no significant difference was observed between each treatment group.

Any remaining treated algae in the digestive tract may lead to overestimation of bioaccumulation. Gillis, Chow-Fraser [112] determined the length of time required to completely depurate metal contaminated sediments from the digestive track of *D*. *magna* in the presence of algae and recommend a minimum of 8 h. Petersen, Akkanen [113] observed a limited depuration of carbon nanotubes from *D*. *magna* after 48 h whereas Zhu, Chang [49] observed complete depuration of food associated TiO_2_ NPs from the gut of *D*. *magna* after 26 h. Following exposure to the treated algae, daphnids were allowed to feed on fresh algae for 72 h in fresh medium and it was assumed all NPs in the digestive track were removed. The algae treatment concentration of AgNP (50 μg L^-1^) was chosen to ensure sufficient Ag concentrations were available for detection in daphnid tissues without causing mortality to algae, based on EC_50_ values obtained through an algae acute test for all types of NPs in a different study (Table B in S1 File). However, since the toxicity of Ag^+^ ions from silver salt (Ag_2_SO_4_) to the alga was very high (72 h EC_50_: 51 μg L^-1^), the feeding and trophic transfer experiments were not conducted for algae contaminated with silver salt at this concentration.

Several other studies have also shown the potential transfer of different types of NPs along the food chain [41, 47, 114]. However, it is not possible to conclude whether Ag was transferred from algae to daphnids in the form of NPs or Ag^+^ ions. McTeer, Dean [42] hypothesized that Ag^+^ ions liberated from AgNPs by dissolution were accumulated by algae and then transferred in to the daphnids. Van Hoecke, De Schamphelaere [90] demonstrated that NPs could not cross the double cell layer of *R*. *subcapitata* when exposed to silica NPs as confirmed by TEM images. Piccapietra, Allué [115] found AgNP internalization was limited when *C*. *reinhardtii* was exposed to carbonate coated AgNPs. However, NPs less than 20 nm may pass through the algal cell walls since the cell walls are porous (5–20 nm in size) and their permeability changes during mitosis. Also, high concentrations of AgNPs may increase the permeability of algae cell wall resulting in more internalization [39]. Miao, Luo [116] confirmed internalization of NPs in the cell after exposing *Ochromonas danica* to AgNPs and Kalman, Paul [39] found AgNP localized in starch granules within the chloroplast of *Chlorella vulgaris* as determined by TEM images. Therefore, the trophic transfer of NPs from algae to daphnids may occur in the forms of NPs or Ag^+^ ions which depend on several factors such as the type of algae, life stage of algae and size of NPs. Data generated from this study clearly show that the type of coating affects the NP accumulation in algae and trophic transfer from algae to daphnids. However, it is not possible to predict the toxicity to daphnids based on bioaccumulation through trophic transfer from this study, and hence, further studies are recommended. The transfer of AgNPs along the aquatic food chain could have adverse implications and therefore there is a need to take this into consideration in protecting aquatic organisms. In doing so, great caution must be taken when assessing the risk of differently coated NPs.

## Conclusion

The type of AgNP coating and medium significantly influenced the degree of aggregation and the behaviour of AgNPs. Based on the 48 h EC_50_ values of *D*. *carinata*, we found that the Ag^+^ ions are significantly more toxic than AgNPs. The toxicity of E-AgNP and C-AgNP were not significantly different, but T-AgNPs were comparatively about 2.5 times less toxic. Since other characteristics such as shape, size and charge are quite similar, the difference in toxicity could be attributed to the effect of different coatings. Feeding experiments revealed that the ingestion rates of NP treated algae were significantly lower than untreated algae revealing associated AgNPs with algae change daphnid feeding behaviour which could have longer term negative effects on *D*. *carinata* population. However, findings from this study are not sufficient to conclude the cause of changed behaviour. Ingestion rates of algae treated with differently coated NPs were not markedly different showing that different types of coatings had little effect on *D*. *carinata* feeding. Our findings also demonstrated the diet-borne transfer of AgNPs from AgNP contaminated *R*. *subcapitata* to *D*. *carinata*. In the algae exposed to AgNPs, T-AgNP bioaccumulation was the highest while C-AgNPs were the lowest. However, bioaccumulation of Ag in daphnids through trophic transfer did not correlate with the accumulation profiles of Ag in algal cells. The percentage Ag retained in daphnids was highest for T-AgNP treated algae while it is lowest for C-AgNPs. These results demonstrate that type of coating may have effects on AgNP accumulation profiles at different trophic levels. The behaviour of differently coated NPs in medium, their toxicity profile and trophic transfer data generated in this study demonstrate the importance of considering type of coating in environmental risk assessment.

## Supporting information

S1 File **Fig A: Chemical formulae**. 1) Tyrosine 2) Epigallecatechin-3-gallate and 3) Curcumin. **Fig B: The SPR of AgNPs in MilliQ water measured after 5 min and 24 h**. (1) T-AgNPs (2) E-AgNPs and (3) C-AgNPs. **Table A: HDD, Zeta potential and PdI of AgNPs measured after 5 min and 24 h**. AgNPs were dispersed in MilliQ water at Ag concentration of 5,000 μg L^-1^. Standard deviations (± SD) are from triplicates. **Table B: 72 h EC_50_ values of AgNPs with different coatings and Ag^+^ ions for algae *Raphidocelis subcapitata***.
(PPTX)
