# Supplementary material for: The toxicity of coated silver nanoparticles to Daphnia carinata and trophic transfer from alga Raphidocelis subcapitata
Source: PLoS One. 2019 Apr 3;14(4):e0214398. doi: 10.1371/journal.pone.0214398 (PMC6447189; doi:10.1371/journal.pone.0214398)
Supplement: S1 File — Fig A: Chemical formulae. 1) Tyrosine 2) Epigallecatechin-3-gallate and 3) Curcumin. Fig B: The SPR of AgNPs in MilliQ water measured after 5 min and 24 h. (1) T-AgNPs (2) E-AgNPs and (3) C-AgNPs. Table A: HDD, Zeta potential and PdI of AgNPs measured after 5 min and 24 h. AgNPs were dispersed in MilliQ water at Ag concentration of 5,000 μg L-1. Standard deviations (± SD) are from triplicates. Table B: 72 h EC50 values of AgNPs with different coatings and Ag+ ions for algae Raphidocelis subcapitata. (PPTX) [file pone.0214398.s001.pptx]

## Slide 1
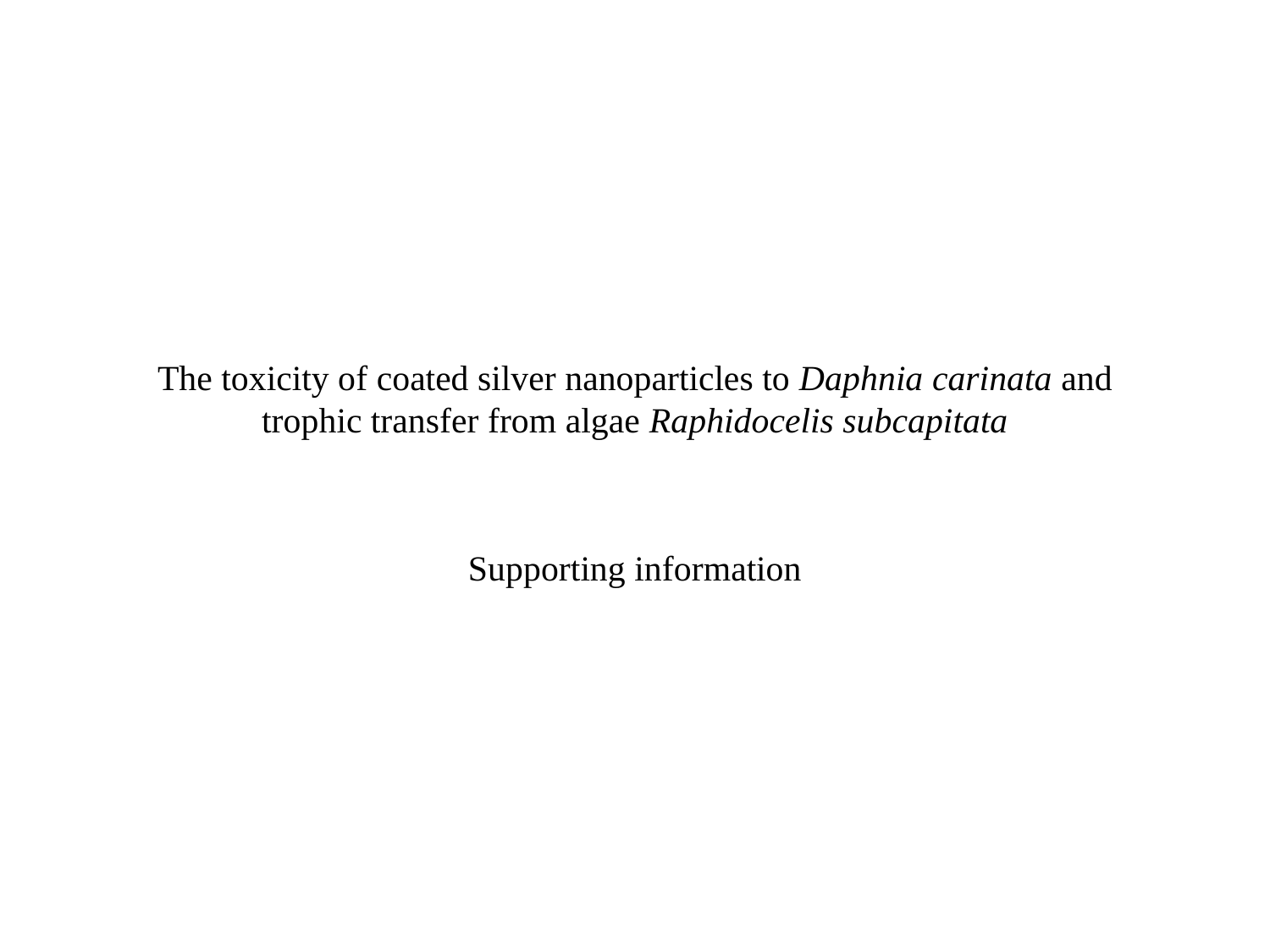

# The toxicity of coated silver nanoparticles to Daphnia carinata and trophic transfer from algae Raphidocelis subcapitata
Supporting information

## Slide 2
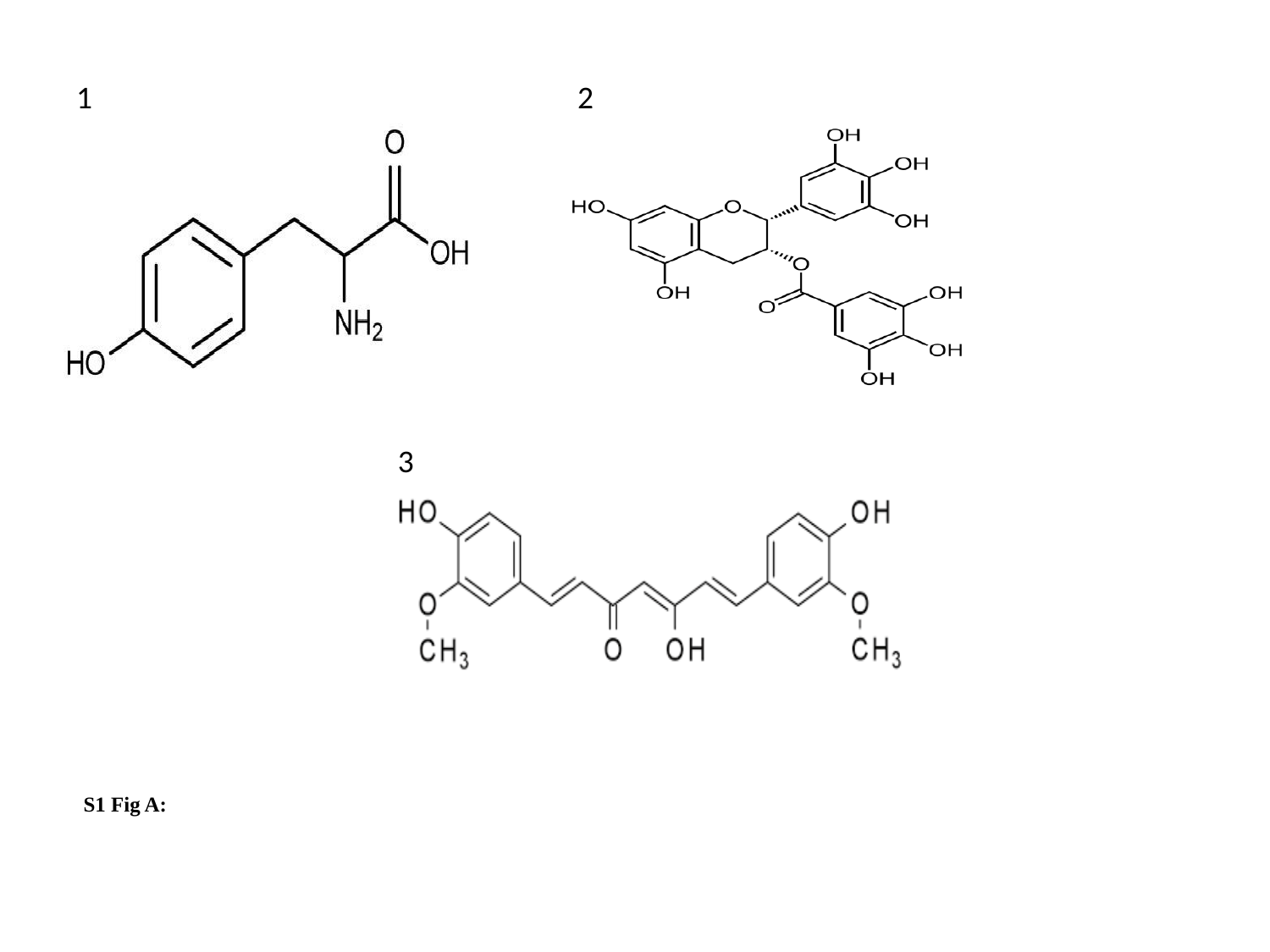

2
1
3
S1 Fig A:

## Slide 3
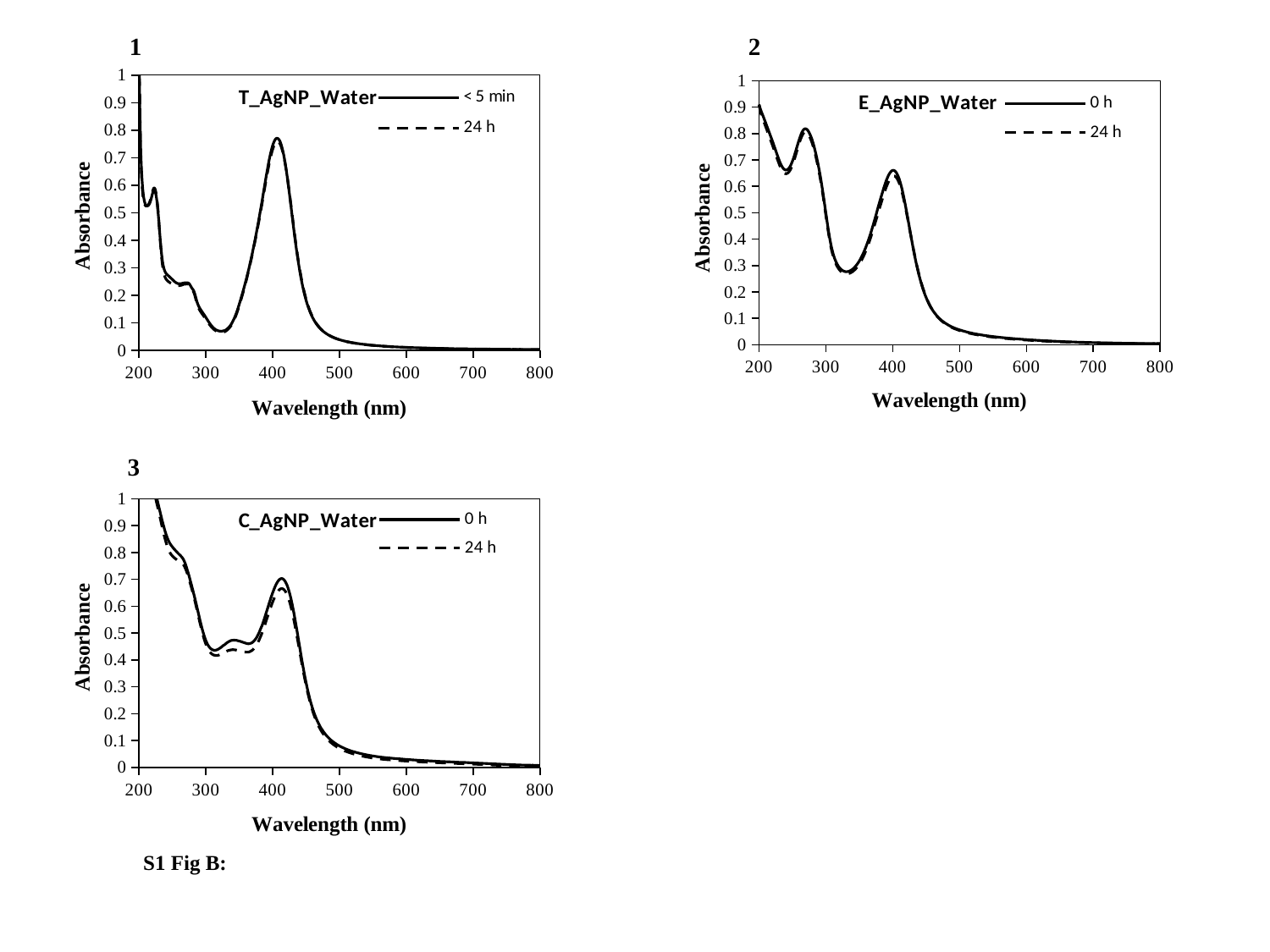

1
### Chart: T_AgNP_Water
| Category | < 5 min | 24 h |
|---|---|---|2
### Chart: E_AgNP_Water
| Category | 0 h | 24 h |
|---|---|---|3
### Chart: C_AgNP_Water
| Category | 0 h | 24 h |
|---|---|---|S1 Fig B:

## Slide 4
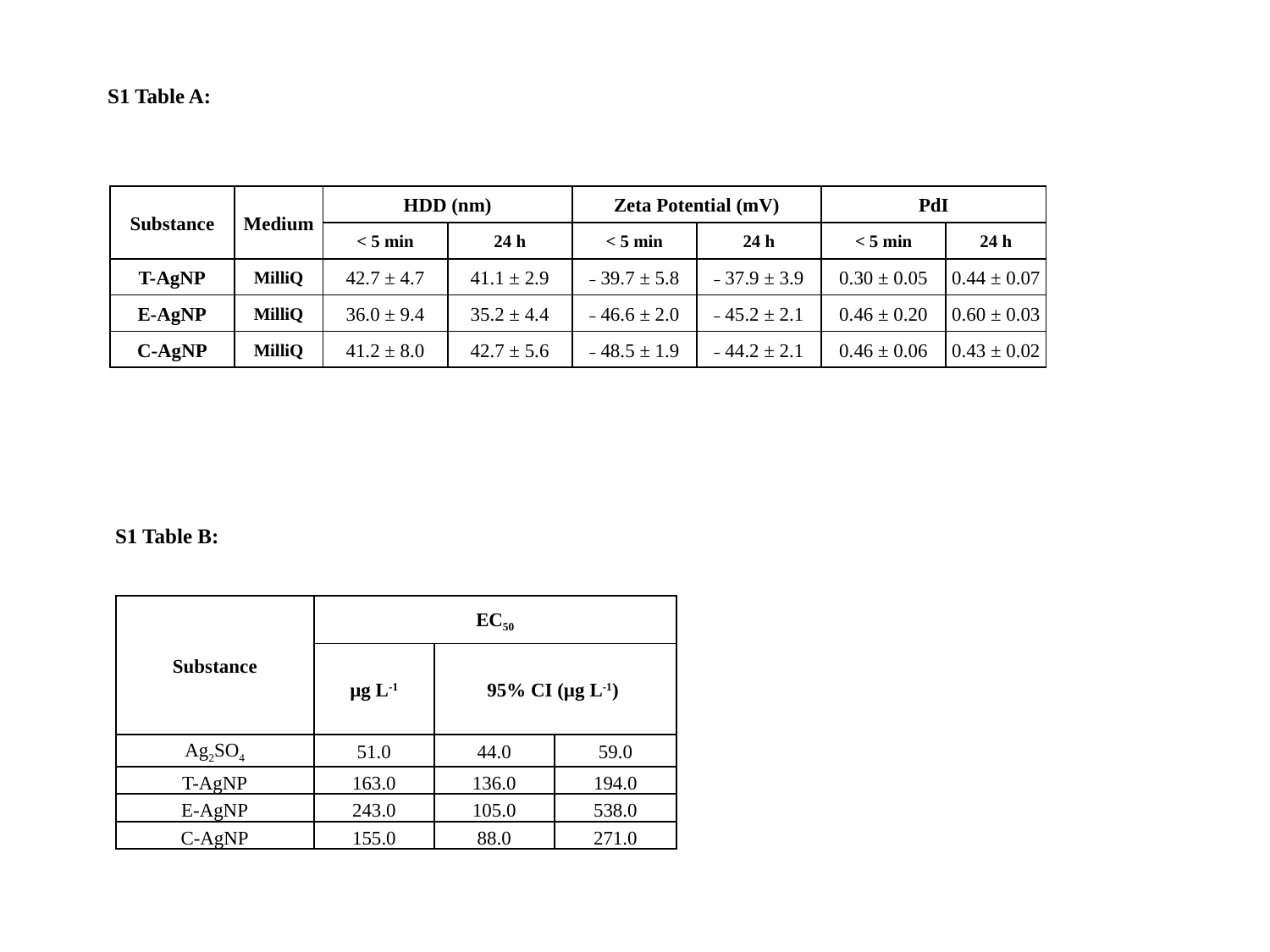

S1 Table A:
| Substance | Medium | HDD (nm) | | Zeta Potential (mV) | | PdI | |
| --- | --- | --- | --- | --- | --- | --- | --- |
| | | < 5 min | 24 h | < 5 min | 24 h | < 5 min | 24 h |
| T-AgNP | MilliQ | 42.7 ± 4.7 | 41.1 ± 2.9 | ˗ 39.7 ± 5.8 | ˗ 37.9 ± 3.9 | 0.30 ± 0.05 | 0.44 ± 0.07 |
| E-AgNP | MilliQ | 36.0 ± 9.4 | 35.2 ± 4.4 | ˗ 46.6 ± 2.0 | ˗ 45.2 ± 2.1 | 0.46 ± 0.20 | 0.60 ± 0.03 |
| C-AgNP | MilliQ | 41.2 ± 8.0 | 42.7 ± 5.6 | ˗ 48.5 ± 1.9 | ˗ 44.2 ± 2.1 | 0.46 ± 0.06 | 0.43 ± 0.02 |
S1 Table B:
| Substance | EC50 | | |
| --- | --- | --- | --- |
| | µg L-1 | 95% CI (µg L-1) | |
| Ag2SO4 | 51.0 | 44.0 | 59.0 |
| T-AgNP | 163.0 | 136.0 | 194.0 |
| E-AgNP | 243.0 | 105.0 | 538.0 |
| C-AgNP | 155.0 | 88.0 | 271.0 |
